# Exploring organizational aspects that promote health-related preventive behavior: using the example of work-related SARS-CoV-2 infection control measures in Germany, August 2020 to November 2021

**DOI:** 10.3389/fpubh.2024.1388996

**Published:** 2024-10-02

**Authors:** Jana Soeder, Anke Wagner, Anna T. Neunhöffer, Peter Martus, Falko Papenfuss, Andrea Wittich, Juliane Schwille-Kiuntke, Esther Rind, Monika A. Rieger

**Affiliations:** ^1^Institute of Occupational and Social Medicine and Health Services Research, University Hospital Tübingen, Tübingen, Germany; ^2^Institute for Clinical Epidemiology and Applied Biometry, University Hospital Tübingen, Tübingen, Germany; ^3^Medical Services, Robert Bosch GmbH, Stuttgart, Germany; ^4^Occupational Psychologist and Psychotherapist, Tübingen, Germany

**Keywords:** COVID-19, SARS-CoV-2, occupational safety and health, workplace, working condition, health promotion, safety culture, attitude

## Abstract

**Introduction:**

During the communicable coronavirus disease (COVID-19) pandemic, organizational infection control measures (oICMs) were introduced in the workplace. The employees’ positive attitudes and active participation are relevant for full effectiveness regarding disease prevention. Therefore, we explore changes in employees’ attitudes toward oICM at work from August–October 2020 (T0) over January 2021 (T1) to October–November 2021 (T2). We further investigate the role an organization can play in supporting health-related preventive behavior.

**Methods:**

We considered repeated cross-sectional and longitudinal panel survey data from 5,554 employees of a global supplier of technology and services in Germany. A total of 16 items constitute the attitude scores toward oICM (5-point Likert scale). Via mixed-effect model, aspects associated with employees’ attitudes toward oICM were explored. Via ‘extreme-group’ approach, we compared the 20% of participants with the largest changes into less favorable to the 20% with the largest changes into more favorable attitudes toward oICM over time.

**Results:**

The overall positive attitudes toward work-related oICM were more favorable at T1 (mean ± SD: 4.2 ± 0.6, median (IQR): 4.3 (0.8), *n* = 2,515) compared to T0 (4.1 ± 0.6, 4.1 (0.8), *n* = 2,417) but less favorable at T2 (3.9 ± 0.7, 4.0 (0.9), *n* = 2,062). Among others, feeling well-informed about possible work-related risks of infection with the severe acute respiratory syndrome coronavirus 2 (SARS-CoV-2), perceived psychosocial demands through work environment aspects, and perceived management’s commitment to safety and health were associated with long-term positive attitudes toward oICM. Individuals developing more favorable attitudes toward oICM reported feeling well-informed about possible work-related SARS-CoV-2 infection risks and improved COVID-19-specific resilience over time. Individuals developing less favorable attitudes toward oICM reported decreased perceptions of COVID-19-associated risks.

**Conclusion:**

oICMs in the workplace were perceived appropriate even after COVID-19 vaccines were widely available although the perceived affective risks about SARS-CoV-2 decreased. Taken together, our findings highlight how organizations can support employees in adopting health-related preventive behavior. Among others, we found that feeling well-informed about possible work-related health risks was positively associated with long-term favorable attitudes toward work-related oICM. We expect that the results contribute to the development of interventions to prepare and adapt to future global public health concerns.

## 1 Introduction

Public health measures for infection control were recommended by, for example, the World Health Organization (WHO) and introduced in the workplace during the severe communicable coronavirus disease (COVID-19) pandemic (1). Temporary behavioral infection control measures (ICMs) were designed to prevent work-related outbreaks from spreading to family, friends, and the public and to prevent healthcare systems from being overwhelmed. This was important because occupational characteristics and workplace conditions were found to impact the work-related risk of infection with the severe acute respiratory syndrome coronavirus 2 (SARS-CoV-2) (2–4).

Governments therefore integrated occupational safety and health (OSH) into their pandemic roadmaps for infection control during the COVID-19 pandemic (1, 5). For example, in Germany, the SARS-CoV-2-Occupational Health and Safety Ordinance was issued to ensure the employees’ protection against infection in the workplace (6). Hence, public health and OSH were closely intertwined in Germany during the COVID-19 pandemic and OSH professionals adapted public health interventions to the organizational context. Workstations were, for example, separated with Plexiglas planes (technical), occupational health services expanded vaccination capacities (organizational), and, in addition, masks were worn (personal measures). According to the hierarchy of prevention and control measures established by the European Agency for Safety and Health for Work (EU-OSHA), technical and organizational take precedence over personal measures (7). Thus, employees’ workability was maintained, return to on-site work safely was allowed, and economic activities were ensured.

The introduced ICMs elicit compulsive changes in daily work: Timeslots for SARS-CoV-2-antigen-testing are considered in work schedules, and meetings are moved online where possible. It is relevant to understand in depth how employees experience these changed working conditions and accept the new working conditions over the long course of the COVID-19 pandemic. This comprehensive understanding helps to derive and adapt appropriate occupational interventions to support long-term health-related preventive behaviors. Although the overall responsibility for introducing, managing, and enforcing mandatory safety and health measures lies with the employer, the employee has a shared responsibility for the appropriate application of these measures. Therefore, the active participation of workers in COVID-19 preventive behaviors, for example, properly conducting rapid tests, is highly relevant to achieve the full effectiveness of ICM and a successful reduction of SARS-CoV-2 transmissions. To investigate how employees improve preventive and suppress unsafe behaviors in the workplace, for example, sun protection in outdoor workers (8), behavioral change theories are applied in OSH research. Those frameworks help to better understand potential barriers and facilitators to or against a certain behavior intention (9). To explore health-related preventive behaviors, for example, adherence to COVID-19-preventive behaviors, behavioral change theories such as the Theory of Planned Behaviors or Social Ecological Model are often applied (9, 10). Here, attitude expresses the extent of an un/favorable evaluation of a particular behavior and is associated with an individual’s preventive behavior intention (11). A lack of research exists in both, public health and OSH, (12) and OSH, to examine interventions aimed at supporting long-term adherence to health-related safety measures.

In our study, we are particularly interested in organizational aspects that promote individuals’ attitudes toward health-related preventive behaviors in the workplace during the COVID-19 pandemic. We address a gap in current knowledge by exploring how organizational aspects, for example, safety culture aspects, affect the individuals’ health-related preventive behavior in the workplace during the COVID-19 pandemic. We expect that the results contribute to improving the organizations’ capacities to adapt to future public health challenges. Given the need, but also the complexity of deriving appropriate interventions or even pandemic preparedness plans, it is important to consider broad contextual factors of organizations, including, for example, the size of the company or the availability of adequate human resources. Therefore, we apply the methodological Organizational Health Services Research (OHSR) approach to consider the course of the pandemic and the non-healthcare organizational context, in which the individual is acting. OHSR studies investigate, among other topics, how the delivery of health services and health-related interventions maintain and improve health while considering the real-world context of organizations (13). During the COVID-19 pandemic, health delivery services were extendedly offered in non-healthcare organizations to maintain and improve employee health, for example, testing and vaccination offers provided by company medical service professionals (14, 15).

To our knowledge, a gap in the literature exists on longitudinal research covering the period after COVID-19 vaccines became widely available and distributed. We primarily aim to examine how the employees’ attitudes toward organizational ICM (oICM) in the workplace changed during three relevant phases of the COVID-19 pandemic in Germany: from autumn 2020 (summer plateau with relaxed restrictions) to January 2021 (strengthened lockdown) to autumn 2021 (majority of the German population vaccinated). We subsequently explore the following: what aspects are associated with long-term attitudes toward oICM throughout the pandemic? And how do those employees with the largest changes into more favorable differ from employees with the largest changes into less favorable attitudes toward oICM?

## 2 Materials and methods

### 2.1 Participants

We considered sub-sample data of an explorative modular mixed-methods study project investigating how companies and employees in Germany dealt with adjusted working conditions due to the introduced SARS-CoV-2-ICM (16, 17). This study was approved by the ethics committee of the Medical Faculty, the University of Tübingen, and the University Hospital of Tübingen in June 2020 (No.: 423/2020BO). Informed consent was obtained from all subjects involved in the study. Participants meeting the following criteria were included: personal informed consent, ≥18 years, employed at one of six company facilities (incl. production sites and office campus) in the German federal states of Bavaria, Baden-Wuerttemberg, and Lower Saxony from a worldwide leading global supplier of technology and services. We report following the STROBE guidelines (18).

### 2.2 Data collection

The online survey was administered three times: 10 August to 25 October 2020 (T0), 12 January to 31 January 2021 (T1), and 15 October to 21 November 2021 (T2). The response rate at T0 (22%) and recruiting strategies are described in detail elsewhere (16).

### 2.3 Study context

#### 2.3.1 Study setting

In addition to quantitative employee survey data, we used the findings of three interview excerpts with managers and company medical service personnel of the company group (19) to describe the real-life context in which we assessed the employees’ attitudes toward oICM. Since we focused on the overall content, we expect no loss of meaning from the authors’ self-translation (20). Qualitative results show that the company group used a digitalized work environment solution and proven communication channels already before the COVID-19 pandemic. In terms of the company’s financial and human resources, the qualitative results further show that at the pandemic’s beginning, disinfectants and protective equipment were produced in-house. This allowed to equip employees and suppliers and thus reduce the risk of supply shortages. In addition, a paid 15-min reduction in shift time was introduced to reduce personal contact during shift handoffs (Supplementary Table S1).

#### 2.3.2 COVID-19 pandemic context in Germany

During the first data collection period, free-of-charge SARS-CoV-2-testing facilities increased in Germany (21). The 7-day incidence increased from 5 at the end of July to 111 cases per 100,000 inhabitants by the end of October 2020 (22). At the end of December 2020, the number of severe courses and hospitalized cases due to COVID-19 disease reached a first peak (22). During the second data collection in January 2021, the start of the nationwide vaccination campaign in Germany and a strengthened lockdown including contact restrictions were realized (21). We conducted the third data collection during the fourth COVID-19 pandemic wave (delta variant of concern). In mid-November 2021, 70.4% of the German population (22) and 93.0% of our participants were vaccinated at least once.

### 2.4 Variables

#### 2.4.1 Attitude toward organizational SARS-CoV-2-ICM in the workplace (dependent variable)

We assume that attitude toward preventive behaviors is less affected by social desirability and reporting bias, compared to self-reported adherence to preventive behaviors, for example, reporting actual behavior frequencies. Attitude, one out of three constructs of the Theory of Planned Behavior model, was previously shown to be positively associated with COVID-19-preventive behavior intention (9). Therefore, we expect that the more positive the attitude, the more likely it is that employees will successfully accept the work-related infection control measures, and the more likely it is that employees will transfer the targeted preventive behaviors into their daily working lives. Technical ICMs, for example, installing Plexiglas planes to separate workstations, are implemented by the employer according to the hierarchy of prevention (7). Contrarily, ICMs that require strong adherence by the individual to be fully effective, for example, holding meetings online or wearing masks, bear risks for practical and motivational hurdles (23). Concerning oICM in particular, we assume that organizations can play a crucial role in supporting employees to successfully transfer oICM to real-world workplace conditions. We asked: ‘How appropriate do you consider the following recommended measures to prevent the spread and infection of the coronavirus in the workplace?’ Employees rated 16 measures on a 5-point Likert scale ranging from “not at all suitable” to “very suitable” (1-5 on a Likert Scale), for example, ‘Meetings are held online where possible/for example, via Skype’ or ‘Consulting offers by the on-site doctor or MED’ (Table 1). Items were defined as oICM according to the hierarchy of prevention (7) by four independent professionals in occupational medicine and health services research. Person-specific mean scores were calculated via mean-across-available-item-approach (1.6% of observations had more than one item missing) (24). A total of 13 observations with all items missing were excluded. High values indicate positive attitudes toward oICM. The internal consistency reliability was good with Cronbach’s *α* = 0.88. Attitudes toward oICM correlated with attitudes toward technical (*r* = 0.7) and personal (*r* = 0.7) ICM.

**Table 1:** Items used to assess the attitude score toward organizational SARS-CoV-2 infection control measures in the workplace.

How appropriate do you consider the following recommended measures to prevent the spread and infection of the novel coronavirus in the workplace? Please evaluate all preventive measures on an organizational level listed below from ‘not at all suitable’ (1) to ‘very suitable’ (5). *Für wie geeignet halten Sie die folgenden empfohlenen Schutz-und Hygienemaßnahmen am Arbeitsplatz, um einer Infektion mit dem neuartigen Coronavirus vorzubeugen? Gar nicht geeignet* (1) *bis sehr gut geeignet* (5).
Ventilate rooms regularly. *Regelmäßiges Lüften von Arbeitsräumen*.
Form fixed, no changing teams in the workplace to reduce contact in person. *Bildung von festen, nicht wechselnden Teams am Arbeitsplatz.*
Home office, if possible. *Wenn möglich, Nutzung von Home-Office-Regelungen.*
Meetings are held online where possible, for example, via Skype. *Wenn möglich, Durchführung von Online-Besprechungen, z.B. über Skype.*
Use rooms only if a physical distance of 1.5 m can be ensured. *Besprechungen nur in Räumen durchführen, wenn Abstandsregel (1,5 m) eingehalten werden kann.*
Reduce usage of the canteen. *Nutzung der Kantine reduzieren.*
Define personally assigned workstations. *Feste Zuordnung der Mitarbeitenden zu einem Arbeitsplatz.*
Define personally assigned work equipment (e.g., mouse/keyboard/tools). *Zuordnung von Arbeitsmitteln für bestimmte Personen (z.B. Maus/Tastatur/Werkzeug).*
Avoid group gatherings in the workplace (e.g., shift handoff via telephone, where possible.) *Zusammentreffen (Gruppenbildung) von mehreren Beschäftigten am Arbeitsplatz vermeiden (z.B. falls möglich: telefonischer Schichtwechsel).*
Avoid unnecessary through traffic in highly frequented areas (e.g., offices, halls, and staircases). *Unnötigen Durchgangsverkehr in stark frequentierten Bereichen vermeiden (z.B. in Büros, Hallen, Treppenhaus).*
Break times are held decoupled. *Pausen zeitlich versetzt abhalten.*
Supervision of compliance with the hygiene rules by supervisor. *Überwachung der Einhaltung der Hygieneregeln durch den/die Vorgesetzte/n.*
Employees at high risk for severe course of the disease are especially protected. *Beschäftigte, die zu Risikogruppen für schwere Verläufe gehören, besonders schützen.*
Consulting offers by the on-site doctor or MED. *Beratung durch Betriebsarzt/Betriebsärztin ermöglichen.*
Install informational signs covering hygiene and rules of conduct in the workplace. *Aufstellen von Hinweisschildern mit Informationen zu Hygiene-und Verhaltensregeln am Arbeitsplatz.*
Communication guide for employees with hygiene and conduct rules in the workplace (e.g., correct usage of mouth–nose covering). *Sicherheitsunterweisungen zu Hygiene-und Verhaltensregeln am Arbeitsplatz (z.B. zum korrekten Auf−/ Absetzen der Mund-Nasen-Bedeckung).*

The original German wording of the items is printed in italics. Each item was rated on a 5-point Likert scale.

#### 2.4.2 Independent variables

Self-reported professional activity: office work means that employees spend most of their working time (> 50%) either on-site or remotely, working in offices of different sizes at screens. “‘Assembly line and manufacturing’ characterizes hands-on factory or quality-control tasks on production parts performed on-site at assembly lines, machines, laboratories, and cleanrooms” (16, p. 4). Factory security service personnel ensure safety on the production site and carry out maintenance work; this also includes firefighters and access control staff at the entrance gates. Company medical service personnel include physicians, ergonomists, and psychologists who perform health risk assessments in the workplace, manage health service delivery from preventive care to medical support in the case of work-related injuries, and are responsible for ensuring safe working conditions. Other professional activities for example include trainees.

Perceived psychosocial demands from aspects of the work environment during the COVID-19 pandemic: score out of three items on unfavorable environmental conditions, for example, noise, inadequate room setup, inadequate workplace equipment, from ‘fully agree’ to ‘strongly disagree’ (1–5 Likert scale) (16, 25).

- Being informed about possible risks of SARS-CoV-2 infection at work: ‘inadequately’–‘very good’ (1–5 Likert scale).
- COVID-19-specific reactance: high values (1–7 Likert scale) reflect the experience of feeling frustrated, annoyed, restricted of freedom, and disrupted due to work-related ICM (26).
- Employee’s rating of the employer’s commitment in OSH related to SARS-CoV-2: very low, low, high, and very high.
- COVID-19-disease perception: score from ‘very low’ to ‘very high’ (1–7 Likert scale) out of two items on the virus’ spread and proximity (26).
- Affective risk perception about SARS-CoV-2 in general: score from ‘very low’ to ‘very high’ (1–7 Likert scale) out of three items on fear, worry, and the virus is something I continuously think about (26).
- Perceived adequacy of media coverage of SARS-CoV-2: ‘too little media attention’–‘media hype’ (1–7 Likert scale) (26).
- Perceived personal susceptibility of SARS-CoV-2 infection: ‘not at all susceptible’–‘very susceptible’ (1–7 Likert scale) (26).
- COVID-19-specific resilience: score out of four items rated from ‘strongly disagree’ to ‘strongly agree’ (1–7 Likert scale). We asked the participants to indicate to what extent they agree with each of the following four statements similar to (26): ‘I find ways to keep going’; ‘I know that I can get through hard times’; ‘I learn important lessons for life’; ‘I am learning ways to cope better next time’. High values reflect the pronounced ability to cope with challenges caused by the pandemic (26).
- Subjectively perceived probability of contracting COVID-19 in the workplace: ‘extremely unlikely’–‘extremely likely’ (1–7 Likert scale).

The full employee survey is described in detail elsewhere (16).

### 2.5 Statistical analysis

We present the study population’s characteristics at each timepoint with number to treat, mean ± SD, and median (interquartile range (IQR)) for continuous measures including Likert scales, and absolute and relative frequencies for categorical variables. All observations were pooled for statistical analysis as the outcome variable did not depend significantly on whether employees participated once, twice, or three times (Kruskal–Wallis tests (KWTs): chi-squared = 7.636, *p* = 0.054 (T0); chi-squared = 7.636, *p* = 0.375 (T1); chi-squared = 7.636, *p* = 0.527 (T2)) (27). Supplementary Table S2 indicates the attitude scores toward oICM at each time point of data collection during the COVID-19 pandemic, differentiated by repeated cross-sectional and longitudinal panel data. Self-generated pseudonymized codes were used for assigning multiple observations to the same individual. Median differences between timepoints were evaluated via the Friedman test. If statistically significant, the Wilcoxon signed-rank test was conducted to further compare differences between two timepoints using the R package rstatix. Statistical significance was set at two-sided *p* < 0.05. We report effect sizes with r < 0.3 (small), r ≥ 0.3 and r ≤ 0.5 (moderate), and r > 0.5 (strong effect) (27).

For multivariate analysis, we imputed missing values for the non-socio-demographic explanatory variables (<5% per timepoint) via predictive mean matching using the following packages: naniar and mice (28). The results were similar when analyzing without observations with missing values. See Supplementary Table S3 for an additional sensitivity analysis, when imputing missing values considering the hierarchical structure of the data, using the package jomo. The results did not differ. Participants of diverse gender were excluded due to the low number (*n* < 10 per timepoint). Following the exploratory approach of our study protocol (17), we performed correlation analysis to investigate associations between each possible explanatory variable with the dependent variable (attitude toward oICM). Mean scores for the dependent variable were non-normally distributed and left-skewed (ceiling effect: 5 as the highest response option). The interquartile range (IQR) of all respondents’ ratings on attitudes toward oICM was 3.7–4.6 (skewness = –0.85). Only for correlation analysis, we mirrored the scale, applied log transformation, and again mirrored the scale to allow interpretation of the results with the initial relationship’s direction [ $x_{transformed}=\ln\left(6-x\right)\times \left(-1\right)$ ; new IQR = –0.84 to –0.36; new skewness = 0.014]. We analyzed our data which have a three-level hierarchical structure with multiple timepoints from employees nested within company facilities using a multilevel linear model (27). Using the maximum likelihood method, mixed-effect models were performed considering fixed effects for timepoint as a categorical variable and random intercepts for company facilities and subjects [ attitude~timepoint+(1| subject)+(1| company facilities) )].

The data were divided into training (2/3 of the person-related observations) and validation (1/3) datasets, stratified for company facilities, age, repeated cross-sectional and longitudinal panel data, and gender using the package splitstackshape. Association between each possible explanatory and the dependent variable was tested in the training data as fixed effect, if reasonable with interaction with time. Thus, we tried to find the optimal model in an exploratory manner in the training set. Following a forward approach for model building using the training dataset, we successively added the explanatory variable with the lowest *p*-value and stopped adding further explanatory variables when statistical significance reached *p* > 0.05 for the latest included. The model was then validated using the validation dataset by considering the regression coefficients of the training model. The samples included in the validation dataset were not considered for model building. In this sense, we work with an independent validation dataset. We report R^2^ for the validation dataset to assess the goodness of fit for the derived model. The packages lme4 and lmerTest were used. We controlled for social desirability (29).

For exploratory research, we performed an ‘extreme-group’ analysis (30) and only considered employees with participation at two consecutive timepoints. Following a distributional approach, we compared the 20% of participants with the largest change into more favorable with the 20% of participants with the largest change into less favorable attitudes toward oICM from T0 to T1, respectively T1 to T2. MWU tests were used for continuous, and Pearson’s chi-square test and Fisher’s exact test for categorical variables. Due to the explorative study design, claims of causality are not possible.

Statistical analyses were performed using R version 4.2.1 (31). The package ggplot2 was used for visualization of results. All packages used for statistical analyses are available on CRAN (Comprehensive R Archive Network, https://cran.r-project.org/web/packages/).

## 3 Results

### 3.1 Sample characteristics, descriptive results, and changes over time

We included 5,554 participants (Table 2; Supplementary Figure S1), 96% (*n* = 5,355) were of German nationality. Within those employees who participated at T0, T1, and T2, the attitudes toward oICM were rather positive and scored 4.1 ± 0.6 (median (IQR): 4.1 (0.8)) at T0, 4.2 ± 0.6 (4.2 (0.7)) at T1, and 4.0 ± 0.6 (4.0 (0.8)) at T2 (5-point Likert scale; *n* = 322). Similarly, in all participants, the attitudes toward oICM were rather positive and reached 4.1 ± 0.6 (4.1 (0.8)) at T0 (5-point Likert scale; *n* = 2,417), 4.2 ± 0.6 (4.3 (0.8)) at T1 (*n* = 2,512), and 3.9 ± 0.7 (4.0 (0.9)) at T2 (*n* = 2,062). See Supplementary Table S2 for the subpopulations’ attitudes toward oICM. The median attitudes toward oICM were statistically significantly different across the timepoints (Friedman test: chi-squared (2)=47.521, *p* < 0.001). The attitudes toward oICM were more favorable at T1 compared to T0 (Wilcoxon signed-rank test: *z* = 105,534; *p* = 0.034; r = 0.08 (small)) but less favorable at T2 with a small effect (*z* = 91,333; *p* < 0.001; *r* = 0.23). The attitudes toward oICM differed statistically significantly between professional activities (KWT: T0:chi-squared = 149.05, *p* < 0.001; T1:chi-squared = 81.083, *p* < 0.001; T2:chi-squared = 101.12, *p* < 0.001). The most positive attitudes toward oICM were reported by company medical service personnel (T0:4.3 ± 0.6 (4.5 (0.8), *n* = 44) and T1:4.4 ± 0.5 (4.6 (0.8), *n* = 38)) and remote working office employees (T2:4.1 ± 0.6 (4.1 (0.8), *n* = 833)). The positive attitudes toward oICM were least pronounced by assembly line (T0:3.7 ± 0.8 (3.7 (1.1), *n* = 337) and T2:3.6 ± 0.9 (3.6 (1.2), *n* = 265)) and factory security service employees (T1:3.8 ± 0.9 (4.0 (1.1), *n* = 80)). The perceived probability of SARS-CoV-2 infection in the workplace was rated as rather unlikely at T0 (mean ± SD: 3.3 ± 1.5 (3.0 (2.0)); 7-point Likert scale; *n* = 2,416), T1 (3.3 ± 1.7 (3.0 (3.0)); *n* = 2,508), and T2 (3.1 ± 1.6 (3.0 (2.0)); *n* = 2,059). The median affective risk perceptions about SARS-CoV-2 in general, were statistically significantly different across the timepoints (Friedman test: chi-squared (2)=45.743, *p* < 0.001). Affective risk perceptions about SARS-CoV-2 in general, were rather high at T0 (4.5 ± 1.2 (4.7 (1.7)); 7-point Likert scale; *n* = 2,412) and T1 (4.7 ± 1.3 (4.7 (1.7)); *n* = 2,507) and decreased to T2 (4.3 ± 1.3 (4.3 (1.7)); *n* = 2,061). The affective risk perceptions about SARS-CoV-2 in general, were higher at T1 compared to T0 (Wilcoxon signed-rank test: *z* = 1055345.405; *p* < 0.001 = 0.034; *r* = 0.08 (small)) but decreased to T2 with a small effect (*z* = 9133310.979; *p* < 0.001; *r* = 0.16).

**Table 2:** Sample characteristics at each time point of data collection during the COVID-19 pandemic.

	T0 (*N* = 2,417)			T1 (*N* = 2,512)			T2 (*N* = 2,062)		
Characteristics	m (sd)	md (IQR)	*n* (%)	m (sd)	md (IQR)	*n* (%)	m (sd)	md (IQR)	*n* (%)
Attitude toward organizational SARS-CoV-2-measures on 5-point Likert scale	4.1 (0.6)	4.1 (0.8)	2,417 (100)	4.2 (0.6)	4.3 (0.8)	2,512 (100)	3.9 (0. 7)	4.0 (0.9)	2,062 (100)
Attitude toward organizational SARS-CoV-2-measures on 5-point Likert scale differentiated per professional activity			2,410 (99.7)			2,502 (99.7)			2,057 (99.9)
Assembly line	3.7 (0.8)	3.7 (1.1)	337 (13.9)	3.9 (0.8)	4.0 (1.2)	318 (12.7)	3.6 (0.9)	3.6 (1.2)	265 (12.9)
Company medical service	4.3 (0.6)	4.5 (0.8)	44 (1.8)	4.4 (0.5)	4.6 (0.8)	38 (1.5)	4.0 (0.9)	4.1 (1.4)	28 (1.4)
Factory security service	3.8 (0.9)	3.8 (1.6)	69 (2.9)	3.8 (0.9)	4.0 (1.1)	80 (3.2)	3.7 (0.7)	3.6 (1.0)	30 (1.5)
Office primarily remote	4.2 (0.5)	4.3 (0.8)	712 (29.5)	4.3 (0.5)	4.3 (0.8)	1,208 (48.1)	4.1 (0.6)	4.1 (0.8)	833 (40.4)
Office primarily on-site	4.1 (0.6)	4.1 (0.8)	1,105 (45.7)	4.1 (0.6)	4.2 (0.8)	723 (28.8)	4.0 (0.6)	4.0 (0.9)	770 (37.3)
Other	3.9 (0.6)	4.0 (0.7)	143 (5.9)	4.1 (0.6)	4.2 (0.6)	135 (5.4)	3.7 (0.7)	3.8 (1.0)	131 (6.4)
Gender			2,408 (99.6)			2,467 (98.2)			2,019 (97.9)
Diverse			9 (0.4)			6 (0.2)			7 (0.3)
Women			757 (31.3)			781 (31.1)			690 (33.5)
Men			1,642 (67.9)			1,680 (66.9)			1,322 (64.1)
Age (years)	44.2 (11.1)	46.0 (18.0)	2,352 (97.3)	44.9 (11.1)	46.0 (18.0)	2,433 (96.9)	45.0 (11.1)	47.0 (17.0)	1,864 (90.4)
18–29			299 (12.4)			265 (10.5)			209 (10.1)
30–39			525 (21.7)			550 (21.9)			388 (18.8)
40–49			614 (25.4)			628 (25.0)			480 (23.3)
50–59			773 (32.0)			795 (31.7)			639 (31.0)
60–70			141 (5.8)			195 (7.8)			148 (7.2)
Education			2,411 (99.8)			2,508 (99.8)			2,053 (99.8)
Higher			1,242 (51.3)			1,341 (53.4)			1,165 (56.6)
Intermediate			842 (34.8)			872 (34.7)			669 (32.4)
Primary			327 (13.5)			295 (11.7)			223 (10.8)
Employees’ rating of the employer’s commitment to OSH			2,412 (99.8)			2,507 (99.8)			2,043 (99.1)
Very high			1,307 (54.1)			1,360 (54.1)			1,552 (75.4)
High			984 (40.6)			1,012 (40.3)			436 (21.1)
Low			110 (4.6)			125 (5.0)			48 (2.3)
Very low			11 (0.5)			10 (0.4)			7 (0.3)
Perceived probability of infection with the SARS-COV-2-virus in the workplace^1^	3.3 (1.5)	3.0 (2.0)	2,416 (99.9)	3.3 (1.7)	3.0 (3.0)	2,508 (99.8)	3.1 (1.6)	3.0 (2.0)	2,059 (99.9)
Information about possible risks of infection in the workplace^2^	4.3 (0.9)	4.0 (1.0)	2,389 (98.8)	4.2 (0.9)	4.0 (1.0)	2,481 (98.8)	4.4 (0.8)	5.0 (1.0)	2,039 (98.9)
Affective risk perception about SARS-CoV-2^1^	4.5 (1.2)	4.7 (1.7)	2,412 (99.8)	4.7 (1.3)	4.7 (1.7)	2,507 (99.8)	4.3 (1.3)	4.3 (1.7)	2,061 (100)
Disease perception^1^	5.1 (1.2)	5.0 (1.5)	2,410 (99.7)	5.4 (1.1)	5.5 (1.5)	2,503 (99.6)	5.3 (1.2)	5.5 (1.5)	2,058 (99.8)
COVID-19-specific resilience^1^	5.4 (1.0)	5.5 (1.3)	2,404 (99.5)	5.2 (1.2)	5.3 (1.5)	2,505 (99.7)	5.4 (1.1)	5.5 (1.3)	2,051 (99.5)

m = mean; sd = standard deviation; md = median; IQR = interquartile range; n = absolute numbers for categorical variables or number to treat for continuous variables; % = relative frequency. * Variable Coding: ^1^ 7-point Likert scale; ^2^ 5-point Likert scale; high values represent strong perception.

### 3.2 Mixed-effect model with a single explanatory variable

Independent variables were tested one by one for statistically significant association with the employees’ attitude toward oICM (dependent variable) (Table 3). Regarding socio-demographics, for example, gender, age, and the personality trait conscientiousness (32) were statistically significantly correlated with the dependent variable. Regarding pandemic-awareness-related variables, for example, disease perception, affective risk perception, and COVID-19-specific resilience were positively associated with favorable attitudes toward oICM (dependent variable). If the virus outbreak was considered a media hype, the attitude toward oICM was less favorable. When examining work-related characteristics, less favorable attitudes toward oICM were associated with working full-time, in changing teams, or in shifts. Office and company medical service personnel reported more positive attitudes toward oICM compared to the assembly line or factory security service employees. Favorably perceived psychosocial demands from aspects of the work environment during the pandemic such as advantageous room setup and availability of working equipment were positively associated with favorable attitudes toward oICM. The more confident employees were that colleagues adhere to distance and hygiene rules, or the higher they rated the employer’s commitment in OSH related to SARS-CoV-2, the more positive the reported attitudes toward oICM. In case the employee felt well-informed about possible SARS-CoV-2-related health risks and threats at work, or in case the employee perceived a low COVID-19-specific reactance due to introduced ICM, the attitudes toward oICM developed even more favorable over time.

**Table 3:** Single explanatory variable as a fixed effect with attitude toward organizational SARS-CoV-2 infection control measures (dependent variable) in training dataset via mixed-effects model: attitude ~ timepoint + (1|subject) + (1|company facilities).

Variable group from the questionnaire	Variable*	Statistics		
		Estimate	SE	*p*-value
Socio-demographic characteristics	Age group **Ref = 18–29** 30–39 40–49 50–59 60–69	0.042 0.065 0.126 0.188	0.021 0.019 0.018 0.024	**0.0247** **<0.001** **<0.001** **<0.001**
	Gender **Ref = Male** Female	0.086	0.016	**<0.001**
	German nationality^A^	−0.016	0.038	0.659
	Household size **Ref = 3–4 person** Alone 2 person >4 person	−0.058 0.046 −0.007	0.024 0.017 0.025	**0.014** **0.007** 0.786
	Living in committed relationship^A^	0.008	0.020	**<0.001**
	Health professional in household^A^	−0.038	0.021	0.071
	Personality trait extraversion^2^	−0.007	0.007	0.330
	Personality trait neurozitism^2^	0.007	0.007	0.359
	Personality trait openness^2^	0.012	0.006	0.057
	Personality trait agreeableness^2^	0.006	0.007	0.409
	Personality trait conscientiousness^2^	0.023	0.007	**0.0013**
	Social desirability of exaggerating positive qualities^2^	−0.023	0.007	**0.0013**
	Social desirability of minimizing negative qualities^2^	−0.002	0.007	0.793
General workplace characteristics	Federal state of work **Ref = Bavaria** Baden-Wuerttemberg Lower Saxony	0.022 0.060	0.032 0.013	0.513 0.175
	Temporary work^A^	0.083	0.068	0.224
	Work in changing teams^A^	−0.063	0.016	**<0.001**
	Shift work^A^	−0.167	0.023	**<0.001**
	Short time work^A^	0.037	0.026	0.150
	Full-time job^A^	−0.084	0.020	**<0.001**
	Fixed-term contract^A^	0.005	0.052	0.922
	Manager^A^	−0.009	0.019	0.611
	Employment at company (years)	0.036	0.008	**<0.001**
	Work experience (years)	0.046	0.007	**<0.001**
	Professional education **Ref = Higher** Intermediate Primary	0.002 −0.011	0.017 0.024	0.883 0.649
	Professional activity **Ref = Office on-site** Office remote work Assembly line Company medical service Factory security service Other	0.039 −0.135 0.111 −0.123 −0.053	0.011 0.016 0.047 0.033 0.020	**<0.001** **<0.001** **0.019** **<0.001** **0.0087**
Workplace characteristics related to the COVID-19 pandemic	Perceived psychosocial demands from aspects of social relations at work during the pandemic^2^	0.015	0.007	**<0.001**
	Perceived psychosocial demands from aspects of the work environment during the COVID-19 pandemic^2^	0.055	0.007	**<0.001**
	Perceived psychosocial demands from aspects of work organization during the pandemic^2^	−0.001	0.007	0.848
	Perceived psychosocial demands from aspects of work content during the pandemic^2^Interaction with timepoint T1Interaction with timepoint T2	0.0370.0200.005	0.0070.0090.009	**<0.0010.029**0.607
				Trust in colleges to keep distance^1^	0.046	0.007	**<0.001**
				Trust in colleges’ adherence to hygiene rules^1^	0.035	0.007	**<0.001**
	Encouraging of colleges to adhere to norm use^A^	0.005	0.008	0.557
	Perceived increased sick leave **Ref = No** Yes Not specified	−0.024 0.034	0.019 0.021	0.214 0.104
	Perceived probability of contracting COVID-19 in the workplace^1^ Interaction with timepoint T1 Interaction with timepoint T2	−0.004 −0.024 0.004	0.007 0.009 0.010	0.569 **0.013** 0.724
	Perceived self-efficacy to avoid an infection in the workplace^1^	0.030	0.008	**<0.001**
	Being informed about possible risks of infection in the workplace^2^	0.059	0.007	**<0.001**
	COVID-19-specific reactance^1^	−0.102	0.007	**<0.001**
	Employees’ rating of the employer’s commitment to OSH **Ref = Very high** High Low Very low	−0.087 −0.145 −0.246	0.009 0.023 0.069	**<0.001** **<0.001** **<0.001**
	Perception of SARS-CoV-2 in general	Disease perception^1^	0.077	0.007	**<0.001**
		Affective risk perception^1^	0.072	0.007	**<0.001**
Perceived adequacy of media coverage^1^		−0.067	0.007	**<0.0011**
Perceived helplessness^1^		0.008	0.007	0.307
Novelty of COVID-19^1^		0.007	0.007	0.381
Scientific knowledge about COVID-19^1^		0.005	0.008	0.516
Perceived personal susceptibility^1^Interaction with timepoint T1Interaction with timepoint T2		0.052−0.021−0.017	0.0070.0090.010	**<0.0010.029**0.097
				Expected severity of infection with SARS-CoV-2^1^	0.071	0.007	**<0.001**
				Perceived probability of contracting COVID-19 in private surroundings^1^	0.014	0.007	0.058
Affiliation to risk group according to Robert Koch Institute for developing severe COVID-19^A^		0.067	0.019	**<0.001**
Frequent contact with individuals from the risk group^A^		0.011	0.016	0.465
Usage of the Robert Koch Institute’s Corona-Warn-App^A^		0.081	0.015	**<0.001**
Knowledge about confirmed cases of infected peers^A^		−0.008	0.009	0.408
COVID-19-specific resilience^1^		0.063	0.008	**<0.001**
SARS-CoV-2-specific antibody test conducted^A^Interaction with timepoint T1Interaction with timepoint T2		−0.0140.0390.021	0.0160.0190.021	0.390**0.038**0.324

* Variable coding: ^1^ 7-point Likert scale; ^2^ 5-point Likert scale; ^A^ ref=0=no. 1 = yes. SE = standard error as measure of uncertainty; ref. = reference category. Variables and items were partly self-developed and partly from prevailing surveys including validated scales (see (16) for further details and additional references). Applied transformation of outcome: ^x^transformed = ln(6 − x) × (−1).

### 3.3 Mixed-effect model with multiple explanatory variables

The optimal model derived from the training dataset is shown in Table 4. The mixed-effect model with multiple explanatory variables showed a statistically significant improved fit over the empty random intercept model. When applying this model to the validation dataset, which is independent from the training dataset, it explained 28% of the total variance in the dependent variable (R^2^). Eleven explanatory variables were statistically significantly associated with the long-term attitude toward oICM. Age, as a socio-demographic characteristic, was statistically significantly positively associated with the long-term attitude toward oICM (dependent variable). Regarding organization-related aspects statistically significantly associated with the long-term attitude toward oICM, we identified the following: professional activity, aspects of the work environment with impact on perceived psychosocial demands during the pandemic, level of information about possible work-related risks of SARS-CoV-2 infection, reactance toward work-related ICM, and how the employees rated the employers’ commitment to OSH related to SARS-CoV-2. We controlled for social desirability (29).

**Table 4:** This mixed-effects model to predict attitude toward organizational SARS-CoV-2 infection control measures as a continuous outcome variable was derived in the training dataset and applied to the validation dataset: attitude ~ timepoint + (1|subject) + (1|company facilities).

Explanatory variable (fixed effect)	Estimate (SE)	*p*-value	Estimate (SE)	*p*-value
Timepoint Intercept (T0) T1 T2	−0.608 (0.017) 0.038 (0.009) −0.062 (0.009)	**<0.001** **<0.001** **<0.001**	−0.612 (0.017) 0.043 (0.009) −0.058 (0.010)	**<0.001** **<0.001** **<0.001**
Age group Ref. = 18–29 30–39 40–49 50–59 60–69			0.006 (0.017) 0.014 (0.017) 0.043 (0.016) 0.083 (0.022)	0.735 0.388 **0.008** **<0.001**
Professional activity Ref. = Office on-site Office remote work Assembly line Company medical service Factory security service Other			0.024 (0.010) −0.070 (0.015) 0.057 (0.042) −0.094 (0.029) −0.027 (0.019)	**0.016** **<0.001** 0.169 **0.001** 0.143
Perceived psychosocial demands from aspects of the work environment during the COVID-19 pandemic^2^			0.018 (0.005)	**<0.001**
Being informed about possible SARS-CoV-2 risks of infection in the workplace^2^			0.023 (0.005)	**<0.001**
COVID-19-specific reactance^1^			−0.052 (0.005)	**<0.001**
Employees’ rating of the employer’s commitment to OSH Ref. = Very high High Low Very low			−0.049 (0.009) −0.067 (0.023) −0.078 (0.066)	**<0.001** **0.004** 0.233
Disease perception^1^			0.029 (0.005)	**<0.001**
Affective risk perception^1^			0.033 (0.005)	**<0.001**
Perceived adequacy of media coverage^1^			−0.017 (0.005)	**<0.001**
Expected severity of a SARS-CoV-2 infection^1^			0.019 (0.005)	**<0.001**
COVID-19-specific resilience^1^			0.036 (0.005)	**<0.001**

SE = standard error as measure of uncertainty; ref. = reference category. * Variable coding: ^1^ 7-point Likert scale; ^2^ 5-point Likert scale. Applied transformation: ^x^transformed = ln(6 − x) × (−1).

### 3.4 “Extreme-group” analysis of within-person changes in attitude toward oICM

A total of 712 employees participated at T0 and T1 (Supplementary Figure S2). The 20% (*n* = 143) with the largest within-person changes into more favorable attitudes toward oICM (mean delta = 0.66; range: 0.44–1.63) reported attitudes toward oICM on average of 3.8 ± 0.5 (T0) and 4.4 ± 0.5 (T1). The 20% (*n* = 143) with the largest within-person changes into less favorable attitudes toward oICM (mean delta = −0.57; range: −2.10 to-0.31) reported attitudes toward oICM on average of 4.3 ± 0.5 (T0) and 3.7 ± 0.6 (T1). Both groups differed not statistically significantly in socio-demographics or work characteristics (Table 5). Affective risk perceptions were lower with a small effect at T0 in participants developing more favorable attitudes toward oICM compared to those participants developing less favorable attitudes toward oICM (T0: 4.2 ± 1.1 vs. 4.5 ± 1.3; MWUT: z = 2.603; *p* = 0.009; r = 0.18; T1: 4.4 ± 1.2 vs. 4.7 ± 1.3). Within both groups, participants felt equally informed about possible work-related risks of infection at T0 (4.3 ± 0.8 vs. 4.3 ± 0.8). At T1, those developing more favorable attitudes toward oICM felt better informed than those developing less favorable attitudes toward oICM with a small effect (T1: 4.4 ± 0.7 vs. 4.0 ± 1.0; MWUT: *z* = 2.766; *p* = 0.006; *r* = 0.22).

**Table 5:** Group comparison of participants with the largest changes in attitudes toward organizational infection control measures (oICMs) from T0 to T1, respectively, T1 to T2.

	T0 to T1					T1 to T2				
	“Extreme group” developing less favorable attitudes toward oICM (*N* = 143)		“Extreme group” developing more favorable attitudes toward oICM (*N* = 143)		*p*-value*	“Extreme group” developing less favorable attitudes toward oICM (*N* = 134)		“Extreme group” developing more favorable attitudes toward oICM (*N* = 134)		*p*-value*
	m	sd	m	sd		m	sd	m	sd	
Attitude toward organizational SARS-CoV-2 infection control measures^2^										
T0	4.3	0.5	3.8	0.5	**<0.001**					
T1	3.7	0.6	4.4	0.5	**<0.001**	4.4	0.4	3.8	0.6	**<0.001**
T2						3.5	0.5	4.3	0.5	**<0.001**
Disease perception^1^										
T0	5.1	1.2	4.9	1.1	0.200					
T1	5.4	1.1	5.4	1.0	0.900	5.5	1.0	5.3	1.0	**0.046**
T2						5.1	1.2	5.1	1.2	0.900
Affective risk perception^1^										
T0	4.5	1.3	4.2	1.1	**0.009**					
T1	4.7	1.3	4.4	1.3	**0.041**	4.7	1.2	4.6	1.2	0.700
T2						4.1	1.2	4.2	1.3	0.500
Information about COVID-19-related risks of infection at work^2^										
T0	4.3	0.8	4.3	0.8	0.522					
T1	4.0	1.0	4.4	0.7	**0.005**	4.4	0.7	4.2	0.9	0.085
T2						4.5	0.7	4.4	0.9	0.831
Covid-19-specific resilience^1^										
T0	5.4	1.0	5.1	1.2	0.900					
T1	5.1	1.2	5.4	1.0	0.059	5.3	1.1	5.0	1.1	**0.032**
T2						5.3	1.1	5.4	1.0	0.400

	*n*	%	*n*	%	*p*-value*	*n*	%	*n*	%	*p*-value*
Gender					0.500					0.400
Diverse	1	0.7	0	0		0	0	0	0	
Women	44	30.3	50	35.0		50	37.3	44	32.8	
Men	98	69.0	93	65 0		84	62.7	90	76.2	
Education					0.500					0.300
Higher	76	53.0	79	55.2		82	61.2	70	52.2	
Intermediate	47	33.0	50	35.0		39	29.1	47	35.1	
Primary	20	14.0	14	9.8		13	9.7	17	12.7	
Professional activity										
Assembly line	18	13.0	15	10.0	0.300	11	8.2	23	17.2	0.130
Medical service	5	3.5	1	0.7		3	2.2	1	0.7	
Factory security service	8	5.6	3	2.1		2	1.5	4	3.0	
Office primarily remote	66	46.0	72	50.0		56	42.0	55	41.0	
Office primarily on-site	38	27.0	42	29.0		56	42.0	49	37.0	
Other	7	4.9	10	7.0		6	4.5	2	1.3	
Manager position	24	17.0	32	22.0	0.200	25	18.7	26	19.4	0.900

Variable coding: ^1^ 7-point Likert scale; ^2^ 5-point Likert scale; high values represent strong perception. m = mean; sd = standard deviation; *n* = absolute numbers; % = relative frequency. * Statistical significance of group differences: Mann–Whitney U-test for continuous or person chi-square test/Fisher’s test for categorical variables.

A total of 571 employees participated at T1 and T2 (Supplementary Figure S2). The 20% (*n* = 134) with the largest within-person changes into more favorable attitudes toward oICM (mean delta = 0.46; range: 0.17–0.63) reported attitudes toward oICM on average of 3.8 ± 0.6 (T1) and 4.3 ± 0.5 (T2). The 20% (*n* = 134) with the largest within-person changes into less favorable attitudes toward oICM (mean delta = −0.84; range: −0.56 to-1.94) reported attitudes toward oICM on average of 4.4 ± 0.4 (T1) and 3.5 ± 0.5 (T2). Participants of both groups differed not significantly regarding socio-demographics or work characteristics (Table 5). Participants developing less favorable attitudes toward oICM reported higher disease perceptions with a small effect at T1 (5.5 ± 1.0 vs. 5.3 ± 1.0; MWUT: *z* = 1.996; *p* = 0.046; *r* = 0.15; T2: 5.1 ± 1.2 vs. 5.1 ± 1.2) but larger absolute decreases to T2 compared to participants developing more favorable attitudes toward oICM. Those participants developing more favorable attitudes toward oICM reported increasing COVID-19-specific resilience (T1: 5.0 ± 1.1; T2: 5.4 ± 1.0), whereas those developing less favorable attitudes toward oICM reported stable COVID-19-specific resilience (T1: 5.3 ± 1.1; T2: 5.3 ± 1.1).

## 4 Discussion

### 4.1 Key findings

This longitudinal analysis of pooled repeated cross-sectional and longitudinal panel data revealed that the attitudes toward organizational SARS-CoV-2-ICM in the workplace were very positive in autumn 2020, during the lockdown in January 2021, and in autumn 2021. Regarding organizational aspects to promote the employees’ COVID-19-preventive behaviors associated with long-term positive attitudes toward oICM, we identified the following: working in the office, perceiving favorable psychosocial demands from aspects of the work environment during the pandemic, feeling well-informed about possible work-related risks of SARS-CoV-2 infection, perceiving low reactance toward ICM, and rating the employers’ commitment in OSH related to SARS-CoV-2 as high. The exploratory ‘extreme-group’ approach revealed that employees feeling well-informed about possible risks of SARS-CoV-2 infection and over time improved COVID-19-specific resilience developed more favorable attitudes toward oICM. Employees with large decreases in disease perception developed less favorable attitudes toward oICM over time.

The great majority of our sample reported positive attitudes toward oICM. Work-related ICMs were thus perceived appropriate even after COVID-19 vaccines were available and even though perceived work-related risks of infection and especially the perceived affective risks about SARS-CoV-2 decreased over time. Our study population rated perceived affective risks about SARS-CoV-2 similarly to employed participants within the German serial cross-sectional COVID-19 Snapshot Monitoring (COSMO) study (4.6 on a 7-point Likert scale ranging from ‘very low’ to ‘very high’ on 27 October 2020 (*N* = 920); 4.7 on 26 January 2021 (*N* = 916); 4.5 on 16 November 2021 (*N* = 930)) (26, 41). The percentage of office employees working primarily remotely was highest in January 2021. At this time, nationally stronger restricted laws for employers to enable remote work if possible were realized in Germany (6). Our results align with the findings of the Virus Watch UK-Cohort study, where the majority of multi-occupational workers, for example, healthcare, managers, or administratives, reported most work-related ICM to be still worthwhile in early 2022 after national restrictions were eased (33). Therefore, oICMs seem important for allowing employees to return to work on-site and feel safe concerning possible work-related risks of infection.

Among others, we identified significant associations between positive attitudes toward oICM and organization-related aspects: perceiving favorable psychosocial demands from aspects of the work environment during the pandemic, the perceived employer’s commitment to OSH related to SARS-CoV-2, and the level of information about possible work-related risks of SARS-CoV-2 infection. In a mixed-methods study (34), it was previously highlighted that effective and transparent communication, for example, regarding organizational safety policies, played a major role in making healthcare personnel feel safe and healthy in their workplaces during the COVID-19 pandemic. It was further previously shown that high socio-economic resources of organizations, the managers’ and co-workers’ attitudes and behaviors, and safety knowledge and motivation importantly contribute to a beneficial safety culture (35). Regarding the implementation and success of work-related health-related interventions, active participation and support by managers, for example, by clearly communicating reasons and consequences, were previously shown to be highly relevant (36). During previous global public health threats, misinformation affected trust in medical professionals and public health authorities and negatively impacted adherence to COVID-19 preventive behavior; this threat is assumed to be exacerbated during the COVID-19 pandemic by the widespread use of social media (37, 38). The multivariate analysis in our study population confirmed positive associations between working in the office or company medical service and long-term positive attitudes toward oICM. This is contrary to the negative association we found in our study population for working at assembly lines with long-term positive attitudes toward oICM. Thus, even within the same company group, we revealed differences between professional activities. For interpretation purposes, it should be noted that the employees of the company medical service were responsible for deriving and adapting the recommended SARS-CoV-2 infection protection measures of the SARS-CoV-2 Occupational Health and Safety Ordinance (6) at the respective workplaces.

The exploratory ‘extreme-group’ approach should be interpreted with caution as it only considers a subgroup of the overall study population. However, it revealed that employees feeling well-informed about possible work-related risks of SARS-CoV-2 infection and over time improved COVID-19-specific resilience developed more favorable attitudes toward oICM. Individuals with large decreases in risk or disease perception developed less favorable attitudes toward oICM over time. Our findings align with previous longitudinal results for a representative German population sample, where decreasing risk perceptions are associated with increasing pandemic fatigue; increasing pandemic fatigue is associated with less adherence to health-related safety measures, for example, wearing masks (12).

Multiple studies having applied behavioral change theories to predict COVID-19 preventive behaviors identified that the ability of individuals to improve COVID-19 preventive behaviors depends, among others, on the individuals’ knowledge about the disease and possible associated risks, and their trust in health authorities (9). Taken together, the framework of behavioral change theories helped us to examine long-term attitudes to COVID-19-preventive behaviors at work and elaborate on the role an organization can play regarding possible interventions. Our findings highlight how organizations could be integrated into future public health campaigns to maintain population health during global public health concerns. They further highlight the importance of preparedness strategies.

### 4.2 Strengths and limitations

One strength is the employee survey study design which covers three relevant phases of the COVID-19 pandemic in Germany. The large and multi-occupational study population further allowed the analysis of intra-and interindividual changes, using repeated cross-sectional data and longitudinal panel data. A major strength of our study is the rapid realization of the first survey period, which was carried out in the early stages of the pandemic. The COVID-19 pandemic constituted a new and unknown situation. Thus, the overall study project was designed as an exploratory multimodal mixed-methods approach. Due to the ongoing collaboration with the examined company group, we received the rare opportunity to continue our study project including a third survey wave in autumn 2021, where the majority of employees and the German population were already vaccinated at least once. Because of the codex of good scientific practice, for example, we recruited the same overall sample at survey wave T1 and T2 compared to T0, we decided to stay with the exploratory study design throughout the complete study project. Considering multiple company facilities in three German federal states allowed considering possible differences in safety cultures and local pandemic situations. The methodological OHSR approach (13) allowed the evaluation of the assessed attitudes toward oICM against the pandemic context and the company’s real-life setting to enhance the findings’ external validity. We assessed attitudes toward oICM of employees in partly critical professional activities (company medical service, managers, and manual workers) but mostly not in critical sectors (non-health-related manufacturing; not entrusted with ensuring basic supply for society) (39). In the present study, we collaborated with a large company group with large financial and human resources. Transferability to smaller companies with restricted resources might therefore be limited. Limitations include the low proportion of participants with non-German nationality. Due to the short period of time between the virus outbreak and the first data collection period, the survey was only developed as an online version and in the German language. Therefore, assembly line employees were less likely to take part in our study than office employees. A limitation of our results is that during the COVID-19 pandemic, it was not possible to observe behavior on-site as part of a non-experimental study. Therefore, our results rely on self-reported data. Sampling bias due to increased participation of motivated employees needs to be considered, but we controlled for social desirability (29).

### 4.3 Future work

In terms of future work, extensive qualitative data in addition to quantitative survey data could provide pluralistic perspectives to holistically understand how implemented infection control measures in challenging situations such as the COVID-19 pandemic affect individuals in their daily work lives. From this, practical take-home messages could be derived to create supportive workplace structures to prevent negative exposure in the workplace, for example, changing psychosocial risks. Following the framework of developing and evaluating complex interventions (40), our findings now contribute to hypotheses development (phase 1: modeling). Those hypotheses in addition to extensive qualitative data analysis on similar research topics could therefore be a starting point for exploring different companies on the same research question or for developing a pilot study to prepare the development of a complex public health intervention in the organizational setting.

## 5 Conclusion

Based on the merged repeated cross-sectional and longitudinal panel data, we present the findings about the individuals’ attitudes toward preventive health behaviors and consider the organizational context and impact of the COVID-19 pandemic. The majority of our study participants reported favorable attitudes toward oICM. It is likely that everyone experienced the ICM in a different way and that implementation of oICM and realization of health-related preventive behaviors to reduce SARS-CoV-2 transmission in the workplace is influenced by other, unidentified factors. Nevertheless, we assume that the majority of employees evaluate the organization’s response to the challenges caused by the pandemic as positive. Our findings suggest that possible future interventions that allow employees to experience supportive workplace structures even during challenging situations and promote health-related preventive behaviors should be adapted to professional activity-specific characteristics. In Germany, public health and OSH measures were intertwined during the COVID-19 pandemic, and OSH professionals adapted public health interventions to the organizational context. We examined the role that organizations can play regarding employees adopting work-related preventive behaviors over the long run of this pandemic. Among others, safety culture components such as feeling well-informed about possible work-related health risks and rating the employer’s commitment to OSH related to SARS-CoV-2 as high were positively associated with long-term favorable attitudes toward work-related oICM. Transparent communication about possible work-related risks as well as the challenges and opportunities of the planned OSH measures appear to improve employees’ attitudes toward the targeted preventive health behaviors. Taken together, our findings provide knowledge of how companies’ responses support the employees’ health-related preventive behaviors. They contribute to the development of future pandemic preparedness strategies in occupational settings to meet unprecedented public health challenges in future.

## Data Availability

The datasets presented in this article are not readily available because of the German national data protection regulations. Requests to access the datasets should be directed to Jana Soeder, jana.soeder@med.uni-tuebingen.de.

## References

[1] World Health Organization (WHO). Considerations for public health and social measures in the workplace in the context of COVID-19: annex to considerations in adjusting public health and social measures in the context of COVID-19; 2020 May 10. Available at: https://www.who.int/publications/i/item/WHO-2019-nCoV-Adjusting_PH_measures-Workplaces-2020.1 (Accessed September 18, 2024).

[2] Reuter M Rigó M Formazin M Liebers F Latza U Castell S. Occupation and SARS-CoV-2 infection risk among 108 960 workers during the first pandemic wave in Germany. Scand J Work Environ Health. (2022) 48:446–56. doi: 10.5271/sjweh.4037 35670286 PMC9888438

[3] European Centre for Disease Prevention and Control (ECDC). COVID-19 clusters and outbreaks in occupational settings in the EU/EEA and the UK: technical report; 2020. Available at: https://www.ecdc.europa.eu/en/publications-data/covid-19-clusters-and-outbreaks-occupational-settings-eueea-and-uk (Accessed September 18, 2024).

[4] Buchan SA Smith PM Warren C Murti M Mustard C Kim JH. Incidence of outbreak-associated COVID-19 cases by industry in Ontario, Canada, 1 April 2020-31 march 2021. Occup Environ Med. (2022) 79:403–11. doi: 10.1136/oemed-2021-107879 35022260

[5] Rondinone BM Valenti A Boccuni V Cannone E Boccuni F Gagliardi D. Global policy responses to the COVID-19 pandemic: results of the ICOH survey. Saf Health Work. (2022) 13:141–7. doi: 10.1016/j.shaw.2022.03.008 35345447 PMC8942434

[6] Bundesministerium für Arbeit und Soziales (BMAS) [German Federal Ministry of Labour and Social Affairs]. SARS-CoV-2-Arbeitsschutzverordnung (Corona-ArbSchV) [SARS-CoV-2 occupational health and safety ordinance]; 2021. Available at: https://www.bundesanzeiger.de/pub/publication/5QH1uegEXs2GTWXKeln/content/5QH1uegEXs2GTWXKeln/BAnz%20AT%2022.01.2021%20V1.pdf?inline [https://www.bundesgesundheitsministerium.de/service/gesetze-und-verordnungen/guv-19-lp/coronaschv/coronaschv-en] (Accessed September 18, 2024).

[7] European Agency for Safety and Health for Work (EU-OSHA). Hierarchy of prevention and control measures; 2022. Available at: https://oshwiki.osha.europa.eu/en/themes/hierarchy-prevention-and-control-measures (Accessed September 18, 2024).

[8] Schilling L Schneider S Görig T Spengler M Greinert R Breitbart EW. “Lost in the sun”-the key role of perceived workplace support for sun-protective behavior in outdoor workers. Am J Ind Med. (2018) 61:929–38. doi: 10.1002/ajim.22905 30175492

[9] Anagaw TF Tiruneh MG Fenta ET. Application of behavioral change theory and models on COVID-19 preventive behaviors, worldwide: a systematic review. SAGE Open Med. (2023) 11:20503121231159750. doi: 10.1177/20503121231159750 37026109 PMC10067469

[10] Frounfelker RL Santavicca T Li ZY Miconi D Venkatesh V Rousseau C. COVID-19 experiences and social distancing: insights from the theory of planned behavior. Am J Health Promot. (2021) 35:1095–104. doi: 10.1177/08901171211020997 34074154 PMC8679169

[11] Ajzen I. The theory of planned behavior. Organ Behav Hum Decis Process. (1991) 50:179–211. doi: 10.1016/0749-5978(91)90020-T

[12] Lilleholt L Zettler I Betsch C Böhm R. Development and validation of the pandemic fatigue scale. Nat Commun. (2023) 14:6352. doi: 10.1038/s41467-023-42063-2 37816702 PMC10564944

[13] Ansmann L Baumann W Gostomzyk J Götz K Hahn U Pfaff H. DNVF-memorandum III – Methoden für die Versorgungsforschung, Teil 4 – Konzept und Methoden der organisationsbezogenen Versorgungsforschung. Kapitel 1 – Definition und Konzept der organisationsbezogenen Versorgungsforschung [DNVF-memorandum III – methods for health services research, part 4 – concept and methods for organizational health services research. Chapter 1– definition and concept of organizational health services research]. Gesundheitswesen. (2019) 81:e64–71. doi: 10.1055/a-0862-0527 30952172

[14] Wagner A Keles K Preiser C Neunhöffer AT Soeder J Schwille-Kiuntke J. Assessing attitudes and participation regarding a pilot COVID-19 workplace vaccination program in southern Germany considering the occupational health perspective-a mixed methods study. Vaccines (Basel). (2023) 11:1082. doi: 10.3390/vaccines11061082 37376471 PMC10304481

[15] Neunhöffer AT Gibilaro J Wagner A Soeder J Rebholz B Blumenstock G. Factors associated with the COVID-19 vaccination status of higher education students: results of an online cross-sectional survey at six universities in southwestern Germany. Vaccines (Basel). (2022) 10:1433. doi: 10.3390/vaccines10091433 36146511 PMC9505187

[16] Soeder J Neunhöffer AT Wagner A Preiser C Rebholz B Montano D. Assessing differences in attitudes toward occupational safety and health measures for infection control between office and assembly line employees during the COVID-19 pandemic in Germany: a cross-sectional analysis of baseline data from a repeated employee survey. Int J Environ Res Public Health. (2022) 20. doi: 10.3390/ijerph20010614 PMC9819385 36612934

[17] Rind E Kimpel K Preiser C Papenfuss F Wagner A Alsyte K. Adjusting working conditions and evaluating the risk of infection during the COVID-19 pandemic in different workplace settings in Germany: a study protocol for an explorative modular mixed methods approach. BMJ Open. (2020) 10:e043908. doi: 10.1136/bmjopen-2020-043908 PMC7677339 33208339

[18] Vandenbroucke JP von Elm E Altman DG Gøtzsche PC Mulrow CD Pocock SJ. Strengthening the reporting of observational studies in epidemiology (STROBE): explanation and elaboration. Epidemiology. (2007) 18:805–35. doi: 10.1097/EDE.0b013e3181577511 18049195

[19] Preiser C Ög E Amperidou O Linder V Wagner A Rieger M. Navigating challenges of the COVID-19 pandemic in leadership. First results from qualitative interviews with leaders in companies in Germany. German medical science GMS publishing house; 2022. Available at: https://www.egms.de/static/en/meetings/dkvf2022/22dkvf216.shtml (Accessed September 18, 2024).

[20] Preiser C Tsarouha E Weltermann B Junne F Seifried-Dübon T Hartmann S. Psychosocial demands and resources for working time organization in GP practices. Results from a team-based ethnographic study in Germany. J Occup Med Toxicol. (2021) 16:47. doi: 10.1186/s12995-021-00336-w 34663355 PMC8522246

[21] Schilling J Buda S Fischer M Goerlitz L Grote U Haas W. Retrospektive Phaseneinteilung der COVID-19-Pandemie in Deutschland bis Februar 2021 [retrospective phasing of the COVID-19 pandemic in Germany until February 2021]. Epidemiologisches Bulletin. (2021) 15:8–17. doi: 10.25646/8149

[22] Robert Koch-Institut (RKI). Coronavirus SARS-CoV-2—Aktuelle Situationsberichte, Wochenberichte und Pandemieradar. Täglicher Lagebericht des RKI zur Coronavirus-Krankheit-2019 (COVID-19). 19. November 2021—aktualisierter Stand für Deutschland. [Current Data, Weekly Reports, and Monitoring on COVID-19 pandemic. Daily Update of RKI on COVID-19 disease on November 19, 2021—current information for Germany]; 2021. Available at: https://www.rki.de/DE/Content/InfAZ/N/Neuartiges_Coronavirus/Situationsberichte/Nov_2021/Archiv_Nov_2021.html (Accessed September 18, 2024).

[23] West R Michie S Rubin GJ Amlôt R. Applying principles of behaviour change to reduce SARS-CoV-2 transmission. Nat Hum Behav. (2020) 4:451–9. doi: 10.1038/s41562-020-0887-9 32377018

[24] Newman DA. Missing Data. Organ Res Methods. (2014) 17:372–411. doi: 10.1177/1094428114548590

[25] Ochsmann E. Längsschnittstudie zu Arbeit und Gesundheit in Zeiten der Corona-Pandemie [longitudinal study on work and health in times of the COVID-19 pandemic]: Institut für Arbeitsmedizin, Prävention und Betriebliches Gesundheitsmanagement [Institute for Occupational Medicine, prevention and occupational health management]; (2020). Available at: https://www.uksh.de/arbeitsmedizin-luebeck/Forschung/Forschungsprojekte.html (Accessed November 23, 2023).

[26] WHO Regional Office for Europe. COVID-19 Snapshot MOnitoring (COSMO standard): Monitoring knowledge, risk perceptions, preventive behaviours, and public trust in the current coronavirus outbreak—WHO standard protocol; (2020) Available at: https://projekte.uni-erfurt.de/cosmo2020/web/ (Accessed September 18, 2024).

[27] Field A Miles J Field Z. Discovering statistics using R. Los Angeles, CA, USA: SAGE (2014).

[28] van Buuren S Groothuis-Oudshoorn K. Multivariate imputation by chained equations in R. J Stat Softw. (2011) 45:1–67. doi: 10.18637/jss.v045.i03

[29] Kemper CJ Beierlein C Bensch D Kovaleva A Rammstedt B. Soziale Erwünschtheit-Gamma (KSE-G) [Social Desirability] ZIS—GESIS Leibniz Institute for the Social Sciences. (2014). doi: 10.6102/zis186

[30] Taris TW Kompier MAJ. Games researchers play—extreme-groups analysis and mediation analysis in longitudinal occupational health research. Scand J Work Environ Health. (2006) 32:463–72. doi: 10.5271/sjweh.1051 17173202

[31] R Core Team. R: A Language and Environment for Statistical Computing; (2021). Available at: https://www.R-project.org/ (Accessed Jun 6, 2023).

[32] Rammstedt B Kemper CJ Klein MC Beierlein C Kovaleva A. Big five inventory (BFI-10) ZIS—GESIS Leibniz Institute for the Social Sciences. The publisher is: ZIS-GESIS. Leipzig-Institut für Sozialwissenschaften. (2014). doi: 10.6102/zis76

[33] Beale S Yavlinsky A Hoskins S Nguyen V Byrne T Fong WLE. Between-occupation differences in work-related COVID-19 mitigation strategies over time: analysis of the virus watch cohort in England and Wales. Scand J Work Environ Health. (2023) 49:350–62. doi: 10.5271/sjweh.4092 37066842 PMC10713985

[34] Siddique S Rice S Bhardwaj M Gore R Coupal H Punnett L. Health care organization policies for employee safety and COVID-19 pandemic response: a mixed-methods study. J Occup Environ Med. (2023) 65:1–9. doi: 10.1097/JOM.0000000000002741 36317257 PMC9835239

[35] Wagner A Schöne L Rieger MA. Determinants of occupational safety culture in hospitals and other workplaces-results from an integrative literature review. Int J Environ Res Public Health. (2020) 17:6588. doi: 10.3390/ijerph17186588 32927758 PMC7559364

[36] Nielsen K Randall R. Opening the black box: presenting a model for evaluating organizational-level interventions. Eur J Work Organ Psy. (2013) 22:601–17. doi: 10.1080/1359432X.2012.690556

[37] Jin SL Kolis J Parker J Proctor DA Prybylski D Wardle C. Social histories of public health misinformation and infodemics: case studies of four pandemics. Lancet Infect Dis. (2024). doi: 10.1016/S1473-3099(24)00105-1 38648811

[38] Ishizumi A Kolis J Abad A Prybylski D Brookmeyer KA Voegeli C. Beyond misinformation: developing a public health prevention framework for managing information ecosystems. Lancet Public Health. (2024). doi: 10.1016/S2468-2667(24)00031-8 PMC11369959 38648815

[39] Eurofound. Job quality of COVID-19 pandemic essential workers: European working conditions telephone survey series Publications Office of the European Union (2023). https://www.eurofound.europa.eu/en/publications/2023/job-quality-covid-19-pandemic-essential-workers (Accessed September 18, 2024).

[40] Campbell M Fitzpatrick R Haines A Kinmonth AL Sandercock P Spiegelhalter D. Framework for design and evaluation of complex interventions to improve health. BMJ. (2000). doi: 10.1136/bmj.321.7262.694 PMC1118564 10987780

[41] Betsch C Korn L Burgard T Gaissmaier W Felgendreff L Eitze S. The four weeks before lockdown during the COVID-19 pandemic in Germany: a weekly serial cross-sectional survey on risk perceptions, knowledge, public trust and behaviour, 3 to 25 march 2020. Euro Surveill. (2021) 26:2001900. doi: 10.2807/1560-7917.ES.2021.26.42.2001900 34676821 PMC8532505

