# Supplementary material for: Exploring organizational aspects that promote health-related preventive behavior: using the example of work-related SARS-CoV-2 infection control measures in Germany, August 2020 to November 2021
Source: Front Public Health. 2024 Oct 2;12:1388996. doi: 10.3389/fpubh.2024.1388996 (PMC11480029; doi:10.3389/fpubh.2024.1388996)
Supplement: Supplementary file 1 [file Data_Sheet_1.pdf]

Supplementary Material

1 Supplementary Data

2 Supplementary Figures and Tables

2.1 Supplementary Tables

Supplementary Table S1. Three interview excerpts are shown here to describe the study setting in which the employee survey (quantitative module) was distributed. The interviews were conducted for the qualitative module of the explorative modular mixed-methods study project (17, 19). In total, eight managers and company medical service personnel were interviewed on the topic of working conditions and work-related infection control measures during the COVID-19 pandemic.

The original German wording.	*The translated English wording.*
„Wir haben teilweise unsere Zulieferer mit Schutzkleidung unterstützt, damit sie nicht geschlossen wurden, sondern uns weiter beliefert haben.“	“We supported our suppliers in some cases with protective equipment so that they did not have to stop their work but could continue to supply us.”
„Aber ansonsten ist das ja in so einem großen Betrieb relativ einfach. Da kann man ja auf viele Ressourcen zurückgreifen. Wir haben zum Beispiel das Desinfektionsmittel selber gemacht, weil wir ein Labor haben, weil es einfach am Markt nicht zu bekommen war, seitdem weiß ich auch, wie man Desinfektionsmittel macht (lacht) und haben das einfach selber angerührt. Wir haben eine Ausbildungsabteilung, die hat dann also erst einmal relativ viel Plexiglas noch auf Lager gehabt und wir haben dann diese Scheiben flächendeckend installiert.“	“But otherwise, it's relatively easy in such a large company. You can draw on a lot of resources. For example, we made the disinfectant ourselves because we have a laboratory, because it simply wasn't available on the market, since then I also know how to make disinfectant (laughs) and simply mixed it ourselves. We have a trainee department, so they first had a relatively large amount of Plexiglas still in stock and we then installed these panes across the company.”
„Also wir arbeiten jetzt zum Beispiel weniger, weil wir eine so genannte Schichtentkopplung haben. Das heißt, die Mitarbeiter hören am Schichtende eine Viertelstunde früher auf, diese Viertelstunde wird aber ganz normal bezahlt.“	“For example, we now work less because we have a so-called shift decoupling. This means that employees stop working a quarter of an hour earlier at the end of their shift, but these fifteen minutes are paid as usual.”

The translation of the quotes was checked by a native speaker during proofreading.

Supplementary Table S2. Attitude scores toward organizational SARS-CoV-2 measures for infection control in the workplace at each time point of data collection during the COVID-19 pandemic differentiated by repeated cross-sectional and longitudinal panel data. [m=mean; sd=standard deviation; md=median; IQR=interquartile range; n=absolute numbers]

Attitude toward organizational SARS-CoV-2 infection control measures on a 5-point Likert scale				
		**T0**	**T1**	**T2**
	n	2417	2512	2062
**All participants**	m (SD)	4.1 (0.6)	4.2 (0.6)	3.9 (0.7)
	md (IQR)	4.1 (0.8)	4.3 (0.6)	4.0 (0.9)
	n	322	322	322
**Employees who participated at T0, T1, and T2**	m (SD)	4.1 (0.6)	4.2 (0.6)	4.0 (0.6)
	md (IQR)	4.1 (0.8)	4.2 (0.7)	4.0 (0.8)
	n	390	390	
**Employees who participated at T0 and T1**	m (SD)	4.1 (0.6)	4.2 (0.6)	
	md (IQR)	4.2 (0.7)	4.3 (0.8)	
	n	154		154
**Employees who participated at T0 and T2**	m (SD)	4.1 (0.6)		4.0 (0.6)
	md (IQR)	4.1 (0.9)		4.0 (1.0)
	n		249	249
**Employees who participated at T1 and T2**	m (SD)		4.2 (0.6)	4.0 (0.6)
	md (IQR)		4.2 (0.8)	4.0 (0.8)
	n	1551	1551	1337
**Employees who participated once**	m (SD)	4.0 (0.7)	4.1 (0.6)	3.9 (0.7)
	md (IQR)	4.1 (0.9)	4.3 (0.9)	4.0 (0.9)

Supplementary Table S3. During the review process, we performed an additional sensitivity analysis when deriving the optimal model from the training dataset. We here imputed the missing values considering the hierarchical structure of the data, using the package jomo.

	**'Null' model**		**'Final' model**	
Explanatory Variable (fixed effect)	Estimate (SE)	p-value	Estimate (SE)	p-value
Timepoint				
Intercept (T0)	-0.608 (0.017)	**<0.001**	-0.583 (0.028)	**<0.001**
T1	0.039 (0.009)	**<0.001**	0.044 (0.009)	**<0.001**
T2	-0.061 (0.009)	**<0.001**	-0.058 (0.010)	**<0.001**
Age group				
ref=18-29				
30-39			0.007 (0.017)	0.683
40-49			0.015 (0.017)	0.370
50-59			0.044 (0.017)	**0.007**
60-69			0.082 (0.022)	**<0.001**
Professional activity				
ref=Office on-site				
Office remote work			0.024 (0.010)	**0.016**
Assembly line			-0.068 (0.015)	**<0.001**
Company medical service			0.062 (0.041)	0.135
Factory security service			-0.093 (0.029)	**0.002**
Other			-0.027 (0.019)	0.154
Perceived psychosocial demands from aspects of the work environment during the COVID-19 pandemic [2]			0.019 (0.005)	**<0.001**
Being informed about possible SARS-CoV-2 risks of infection in the workplace [2]			0.022 (0.005)	**<0.001**
COVID-19-specific reactance[1]			-0.052 (0.005)	**<0.001**
Employees' rating of the employer's commitment to OSH				
ref = Very high				
High			-0.050 (0.009)	**<0.001**
Low			-0.070 (0.023)	**0.002**
Very low			-0.079 (0.066)	0.227
Disease perception[1]			0.031 (0.005)	**<0.001**
Affective risk perception[1]			0.033 (0.005)	**<0.001**
Perceived adequacy of media coverage[1]			-0.016 (0.005)	**<0.001**
Expected severity of a SARS-CoV-2 infection[1]			0.020 (0.005)	**<0.001**
COVID-19-specific resilience[1]			0.037 (0.004)	**<0.001**

* Variable coding: [1] 7-point Likert scale; [2] 5-point Likert scale

Applied transformation: $x_{transformed} = \ln(6 - x) \times (-1)$

2.2 Supplementary Figures

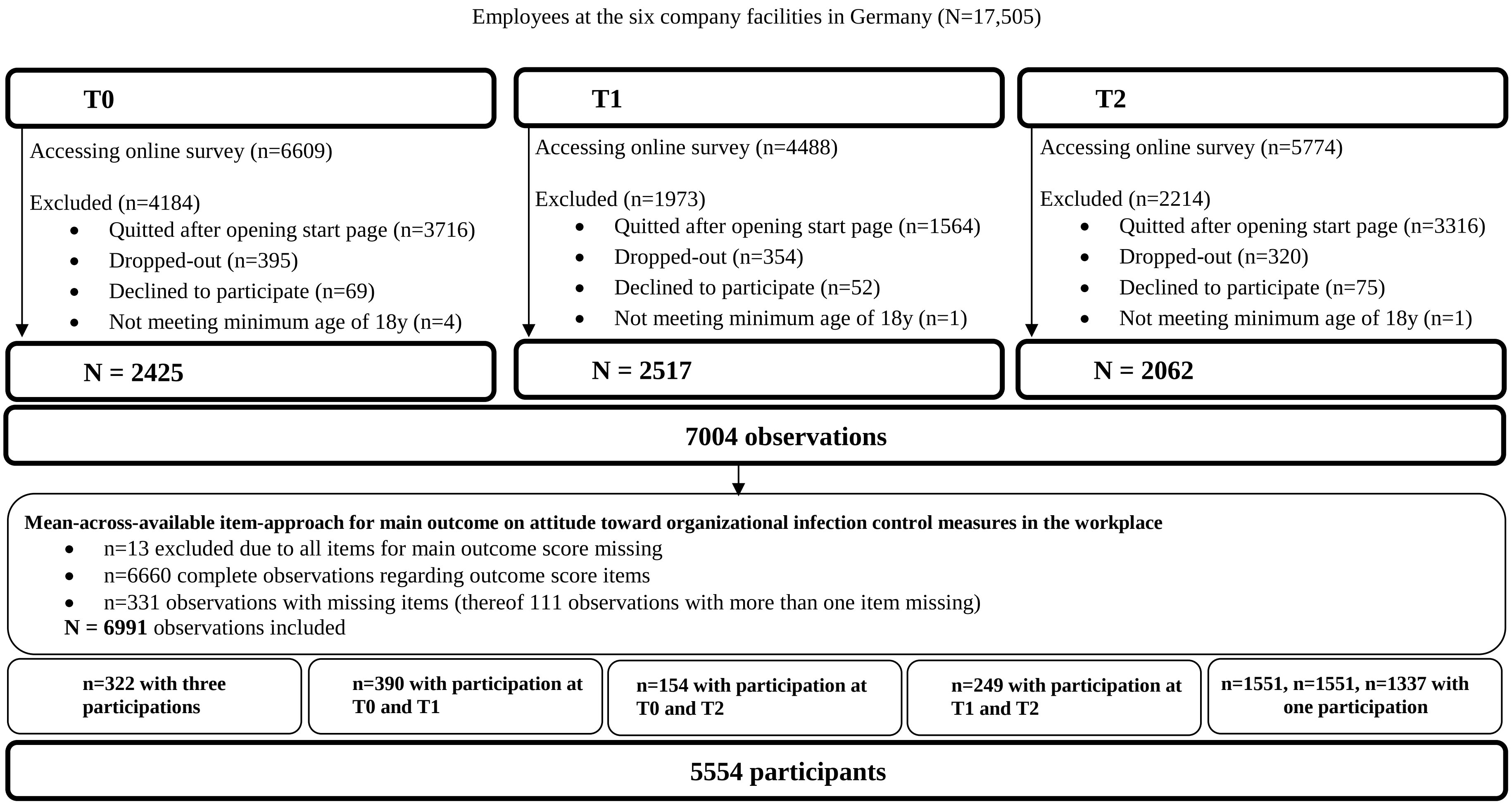

Supplementary Figure S1. Consort Flow Diagram

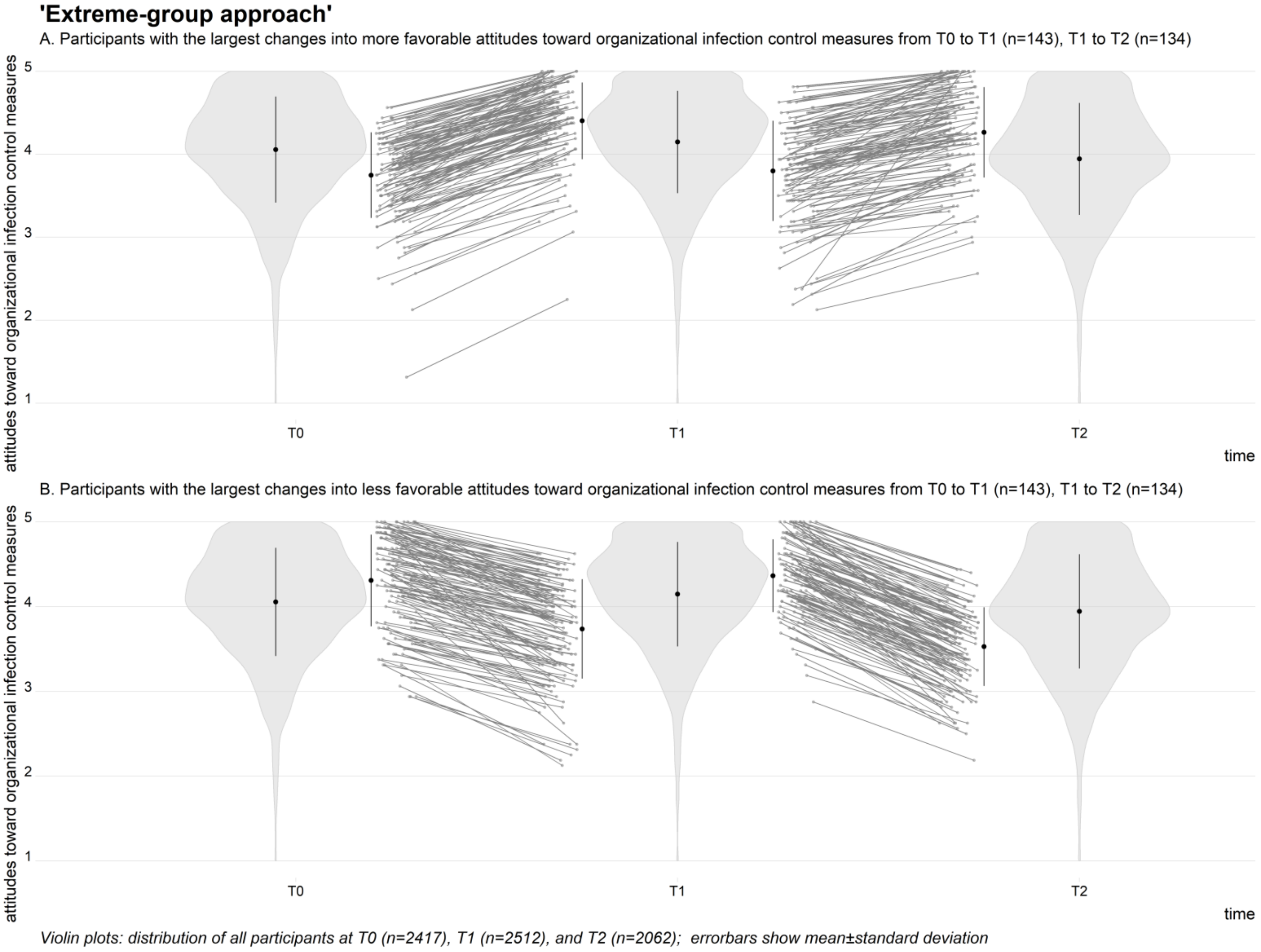

Supplementary Figure S2. ‘Extreme-group’ visualization. The within-person differences of participants with the largest changes in attitudes toward organizational infection control measures are plotted against the overall distribution.
